# Components of the Endosome-Lysosome Vesicular Machinery as Drivers of the Metastatic Cascade in Prostate Cancer

**DOI:** 10.3390/cancers17010043

**Published:** 2024-12-26

**Authors:** Bukuru Dieu-Donne Nturubika, Jessica Logan, Ian R. D. Johnson, Courtney Moore, Ka Lok Li, Jingying Tang, Giang Lam, Emma Parkinson-Lawrence, Desmond B. Williams, James Chakiris, Madison Hindes, Robert D. Brooks, Mark A. Miles, Stavros Selemidis, Philip Gregory, Roberto Weigert, Lisa Butler, Mark P. Ward, David J. J. Waugh, John J. O’Leary, Douglas A. Brooks

**Affiliations:** 1 Clinical and Health Sciences, University of South Australia, Adelaide, SA 5000, Australia; jessica.logan@unisa.edu.au (J.L.); courtney.moore@unisa.edu.au (C.M.); ka_lok.li@mymail.unisa.edu.au (K.L.L.); jingying.tang@unisa.edu.au (J.T.); emma.parkinson-lawrence@unisa.edu.au (E.P.-L.); des.williams@unisa.edu.au (D.B.W.); james.chakiris@mymail.unisa.edu.au (J.C.); madison.hindes@mymail.unisa.edu.au (M.H.); rob.brooks@unisa.edu.au (R.D.B.); 2 Centre for Cancer Biology, University of South Australia, Adelaide, SA 5000, Australia; giang.lam@unisa.edu.au (G.L.); philip.gregory@unisa.edu.au (P.G.); david.waugh@unisa.edu.au (D.J.J.W.); 3 Centre for Respiratory Science and Health, School of Health and Biomedical Sciences, RMIT University, Bundoora, VIC 3083, Australia; mark.miles@rmit.edu.au (M.A.M.); stavros.selemidis@rmit.edu.au (S.S.); 4 Laboratory of Cellular and Molecular Biology, Center for Cancer Research, National Cancer Institute, National Institutes of Health, Bethesda, MD 20892, USA; weigertr@mail.nih.gov; 5 South Australian ImmunoGENomics Cancer Institute, Freemasons Centre for Male Health and Wellbeing, University of Adelaide, Adelaide, SA 5000, Australia; lisa.butler@adelaide.edu.au; 6 Solid Tumour Program, Precision Cancer Medicine Theme, South Australian Health and Medical Research Institute, Adelaide, SA 5000, Australia; 7 Department of Pathology, The Coombe Women and Infants University Hospital, Trinity College Dublin, D08 XW7X Dublin, Ireland; wardm6@tcd.ie; 8 Department of Histopathology, Trinity College Dublin, D08 XW7X Dublin, Ireland; olearyjj@tcd.ie

**Keywords:** prostate cancer, metastasis, prognosis, biomarkers, endosome-lysosome dynamics, Rabs, molecular motors, vesicular trafficking machinery

## Abstract

Prostate cancer remains a significant global health concern, with over 1.4 million new cases diagnosed and more than 330,000 deaths each year. The primary clinical challenge that contributes to poor patient outcomes involves the failure to accurately predict and treat at the onset of metastasis, which remains an incurable stage of the disease. This review discusses the emerging paradigm that prostate cancer metastasis is driven by a dysregulation of critical molecular machinery that regulates endosome-lysosome homeostasis. Endosome and lysosome compartments have crucial roles in maintaining normal cellular function but are also involved in many hallmarks of cancer pathogenesis, including inflammation, immune response, nutrient sensing, metabolism, proliferation, signalling, and migration. Here we discuss new insight into how alterations in the complex network of trafficking machinery, responsible for the microtubule-based transport of endosomes and lysosomes, may be involved in prostate cancer progression. A better understanding of endosome-lysosome dynamics may facilitate the discovery of novel strategies to detect and manage prostate cancer metastasis and improve patient outcomes.

## Simple Summary

Prostate cancer is a major global health issue, with over 1.4 million new cases and more than 330,000 deaths annually. The main challenge in treating prostate cancer is predicting and managing metastasis, which is currently incurable. This review highlights that metastasis may be driven by disruptions in the vesicular trafficking machinery that controls endosome-lysosome biology. Understanding how changes in the transport of endosomes and lysosomes contribute to cancer progression could lead to new ways to detect and treat metastatic prostate cancer, improving patient outcomes.

## 1. Background

Prostate cancer is one of the most prevalent malignancies affecting men, and globally this involves the diagnosis of over 1.4 million new cases and >330,000 patient deaths each year [1,2,3]. Metastases and castration resistance are directly associated with the significant mortality from prostate cancer and represent critical turning points in the disease course that impact adversely on patient outcomes [4]. Treatment intervention with androgen receptor-targeted agents, taxanes, immunotherapy, targeted radiotherapy, and bone-targeted therapy in most cases only marginally improves survival for patients with advanced disease, highlighting a need to further identify mechanisms underlying prostate cancer metastasis, which can then be targeted. However, current risk stratification strategies have significant problems with accuracy for identifying those patients who will progress to have metastatic disease [4,5,6].

**Figure 1:**
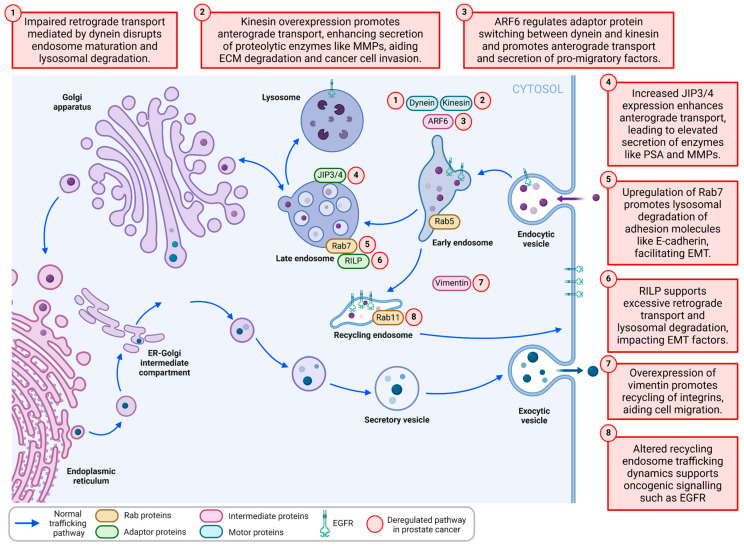
Alterations in the endosome-lysosome system may be key drivers of prostate cancer metastasis.

Alterations to the endosome-lysosome system have been reported to drive key biological features of prostate disease progression (Figure 1). This is presumably due to the central role of these organelles in immunity/inflammation, cell growth, nutrient sensing/recycling, intracellular signalling, intercellular communication, and cell migration [7,8,9]. Recognising the critical contribution of endosome-lysosome function to all the classical hallmarks of cancer biology was the underlying premise for the breakthrough discovery of endosomal biomarkers that define the primary pathogenesis in prostate cancer [8,10,11,12]. A set of endosome-lysosome biomarkers was identified that enable more accurate grading of prostate cancer tissue and prediction of patient biochemical and clinical recurrence/metastasis [8,11,12,13,14,15,16]. The high specificity of these tissue diagnostics suggests that endosomal-lysosomal biology is directly involved in both prostate cancer onset and progression. In this review, we take this a further step and discuss how alterations to vesicular trafficking machinery may regulate endosome-lysosome function and dynamics during prostate cancer progression and metastasis. We also summarise the potential to target this vesicular machinery for biomarker discovery and drug target identification to help address key clinical management problems for patients with prostate cancer metastasis.

## 2. Dysregulation of Endosome-Lysosome Homoeostasis May Promote Prostate Cancer Metastasis

Two main hypotheses are generally accepted to describe the metastatic process, both of which converge on the acquisition of a metastatic phenotype (see [17,18] for a review of both hypotheses). Briefly, the phenotypic plasticity model hypothesises that cancer cells undergo molecular rearrangements that are essential for these cancer cells to leave the primary tumour site, and this results in epithelial-to-mesenchymal transition (EMT) and a more migratory and invasive phenotype. The second clonal model of metastasis hypothesises that a subpopulation of cancer cells is genetically predisposed to metastasis and that driver or acquired mutations endow cancer cells with metastatic potential. These attributes are not mutually exclusive, and it has become increasingly evident that both genetic and non-genetic alterations cooperatively mediate metastatic progression [19]. In addition, alterations to the endosome-lysosome system have been shown to facilitate cancer cell plasticity and activate signalling cascades that promote proliferation, migration, and invasion of metastatic prostate cancer cells [20]. However, the contribution of the endosome-lysosome system to prostate cancer metastasis is less well-defined [21].

Functional alterations of endosomes and lysosomes in prostate cancer cells often feature concomitant disruption to organelle positioning, suggesting that the vesicular trafficking machinery transporting these compartments may be utilised during prostate cancer metastasis [8,22]. For instance, changes in the spatial-temporal dynamics of endosome and lysosome trafficking could lead to increased secretion of proteases that degrade the extracellular matrix, facilitating cancer cell invasion (Figure 1) [23]. Additionally, altered endosome and lysosome function could impact the recycling of cell surface receptors, influencing cell signalling and promoting cell survival, particularly in foreign microenvironments [24]. Thus, the role of vesicular trafficking machinery and how altered organelle dynamics might contribute to prostate cancer progression and metastasis is an attractive concept.

**Figure 2:**
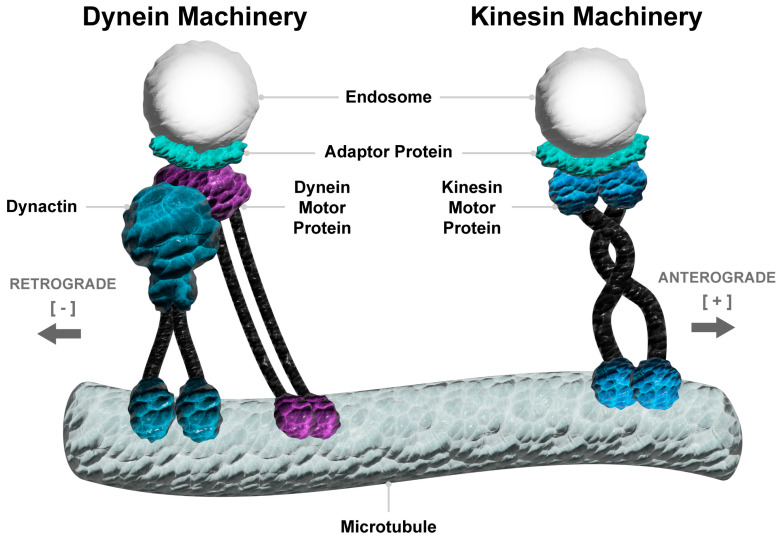
The movement of endosomes and lysosomes on microtubules requires dynein and kinesin motor proteins. Specific adaptor proteins recruit vesicular compartments to dynein and kinesin motor complexes to mediate retrograde movement towards microtubule minus and plus ends, respectively, using ATP hydrolysis as an energy source to drive the process. The dynein motor complex requires stabilisation from the co-factor dynactin.

A multitude of adaptor and scaffolding protein complexes are involved in recruiting specific vesicular trafficking machinery to endosome-lysosome compartments to enable effective organelle transport and positioning (reviewed in [25,26,27,28,29]). For example, the assembly of molecular complexes containing the motor proteins dynein or kinesin enables the bidirectional movement (respectively, retrograde vs. anterograde transport) of endosomes and lysosomes along microtubules, utilising ATP hydrolysis to fuel the transport (Figure 2). The molecular motor dynein is a large multiprotein complex that requires stabilisation from its co-factors to engage endosome-lysosome adaptor proteins to mediate retrograde transport and maturation of endosomes to lysosomes [28,30]. Conversely, there are 15 families of the kinesin molecular motor proteins that are selectively recruited by specific adaptor proteins to mediate anterograde vesicular transport, although several classes of these motor proteins can also mediate retrograde transport [31]. In prostate cancer, a mispositioning of endosome compartments has been observed, which is indicative of altered endosomal biogenesis and maturation and may culminate from dysregulated dynein- or kinesin-mediated retrograde or anterograde transport [8]. Despite this circumstantial evidence for the altered spatial-temporal organisation of endosomes and lysosomes in prostate cancer, the altered regulation, bidirectional transport, and functional impact of this dynamic process are yet to be fully investigated in the context of prostate cancer metastasis.

Adaptor protein complexes can be shared between dynein and kinesin and require regulatory proteins to facilitate the switch between dynein-mediated retrograde and kinesin-mediated anterograde transport [32]. Dysfunction of regulatory proteins and adaptor protein recruitment may lead to impaired endosome trafficking events that promote the metastasis of cancer cells. For instance, the increased expression of the regulatory small GTPase (ADP-ribosylation factor 6) ARF6 in cancer cells leads to the disengagement of the dynein adaptor complex, which is comprised of JNK-interacting protein (JIP)-3 and -4 adaptor proteins. This disengagement of dynein promotes the recruitment of kinesin to mediate anterograde transport of late endosomes and subsequent secretion of metalloproteinases that degrade the extracellular matrix during cancer metastasis [32,33]. While the endosomal release of proteolytic factors such as prostate-specific antigen (PSA) is supported by changes in myosin motor protein expression, which regulates cargo transport on actin cytoskeletal filaments proximal to the plasma membrane, it may also be modulated by microtubule-based anterograde transport, as both systems operate on Rab27 endosomes that transport PSA [34,35,36]. Moreover, changes in expression of components of the microtubule vesicular trafficking system, which control the switch between anterograde and retrograde transport, such as increased ARF6 and JIP4 (encoded by SPAG9), have been reported to support the proliferation and invasion of prostate cancer cells, possibly by promoting endosome anterograde transport, which supports the release of proteolytic factors like PSA [34,35,37,38]. Therefore, an aberrant shift in the dynamic balance between retrograde and anterograde vesicular transport is a potential contributor to prostate cancer metastasis. The known and theoretical components of microtubule vesicular trafficking machinery that may be involved in prostate cancer metastasis are summarised in Table 1.

## 3. Migration and Tissue Invasion by Cancer Cells from the Primary Tumour Within the Prostate

**Figure 3:**
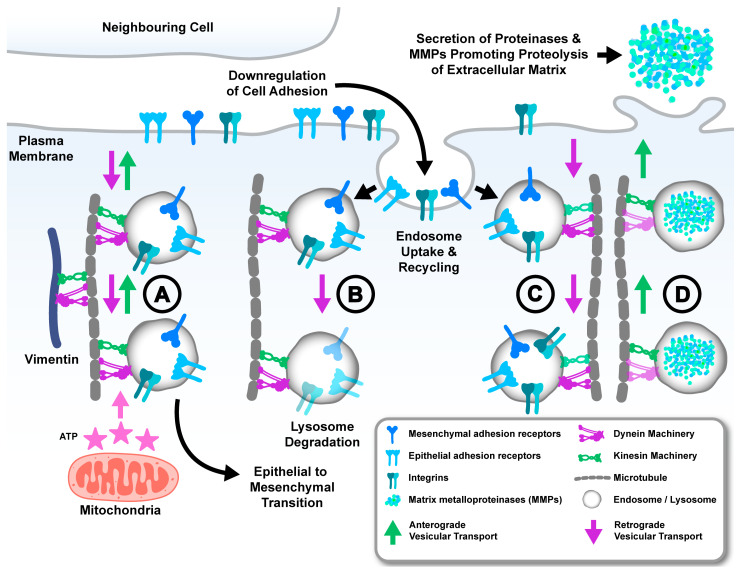
Abnormal localisation and kinetics of endosomes and lysosomes mediated by defective bidirectional transport may aid prostate cancer metastasis via several mechanisms. (A) The dysregulated anterograde and retrograde transport of endosome-lysosome compartments may promote dynamic changes to EMT proteins that facilitate cancer cell detachment and invasion. The metabolic status of the cell may alter the concentration of ATP, which may affect motor protein recruitment to endosomes and impact on ATP-hydrolysis driven processivity to alter endosome bidirectional transport. (B) (degradation) and (C) (internalisation/intracellular traffic). Retrograde transport by dynein trafficking machinery may promote downregulation of integrin proteins to support prostate cancer cell migration. (D) Anterograde transport supported by kinesin trafficking machinery may promote lysosome and endosome secretion of proteolytic factors such as metalloproteinases (MMP) to modify the extracellular matrix (ECM) and support detachment of prostate cancer cells.

Activation of EMT by cancer cells during the initiation of metastasis results in a shift towards a mesenchymal phenotype. This EMT shift is characterised by the selective, temporal, and reversible dysregulation of adhesion receptors, cytokeratins, and mesenchymal intermediate filaments, which facilitate detachment from the primary tumour and enhance cell migration [67,68,69,70]. Partial activation of EMT enhances the metastatic potential of prostate cancer cells and is influenced by transcription factors, cytokines, hypoxia, and androgen signalling. This suggests that an intricate level of plasticity may be operating to exploit endosome and lysosome trafficking and that this is dynamically regulated together with EMT [67,71,72,73,74,75,76]. For example, the observation of high protein expression of the epithelial adhesion receptor E-cadherin in the presence of low mRNA in prostate cancer cells indicates post-translational regulation that may involve a dysfunction of the endosome machinery that regulates the turn-over of these proteins, as observed in other cancers such as colorectal cancer [77,78]. Moreover, analysis of high-grade prostate cancer tissue showed a redistribution of E-cadherin expression from the plasma membrane to the cytosol when compared to low-grade prostate cancers, which is consistent with an altered regulation of E-cadherin recycling or endosome delivery to the plasma membrane (Figure 3A) [79]. This concept is further supported by the observation that the overexpression of Rab11, which regulates slow endosome recycling kinetics, results in increased sequestration of E-cadherin in recycling endosomes and reduces cell surface delivery in HeLa, HT29, and MDCK cells [77,80]. This process may involve alterations in anterograde transport of recycling endosomes, as the depletion of kinesin-3 motor protein impedes the formation of Rab11 recycling endosomes and promotes the generation of enlarged endosomes that sequester cargo [49,50]. Overexpression of wildtype and constitutively active Rab11 promotes the retrograde transport and perinuclear positioning of recycling endosomes [81]. However, the overexpression of a dominant negative mutant promotes anterograde transport and peripheral accumulation of recycling endosomes [81]. This suggests that recycling endosome function, which is critical for the regulation of epithelial adhesion proteins, is modulated by the bidirectional transport of these organelles. Further exemplifying the importance of endosome trafficking kinetics in modulating EMT factors is the observation that the induction of EMT in MDCK cells resulted in E-cadherin accumulation in enlarged vesicles wherein Rab7 activation promoted E-cadherin lysosomal degradation [82,83]. Rab7 activation is known to be sustained by the Rab-interacting lysosomal protein RILP, which concomitantly recruits the dynein motor protein to promote maturation of late endosomes to lysosomes. This suggests that alterations to retrograde transport that mediate endosome maturation may also contribute to E-cadherin dysregulation [84]. Indeed, the inhibition of Rab7 results in a redistribution of E-cadherin-containing endosomes to the cell periphery and promotes E-cadherin recycling, an event that is consistent with attenuated retrograde endosome transport [83]. We have recently reported the upregulation of EMT proteins, including E-cadherin, and decreased cell migration of prostate cancer cells upon knockdown of dynein light intermediate light chain, possibly due to altered lysosome degradation of EMT factors, which may be modulated by dynein-driven retrograde lysosome trafficking [53,85]. These data also suggest that alterations to the trafficking of E-cadherin may involve disrupted activation of Rab proteins, leading to dysregulation of endosome bidirectional transport. This may have consequences for prostate cancer cells because they have increased levels of Rab GDP dissociation inhibitor, which affects the activation of Rab proteins and would therefore influence the endosome trafficking of epithelial adhesion receptors [86]. These changes in endosome kinetics may also be exploited by cancer cells to modulate the expression of mesenchymal adhesion receptors. For example, the inhibition of N-cadherin recycling can also be induced by reducing recycling endosome populations [87,88]. Interestingly, N-cadherin overexpression is increased with androgen receptor inhibition in prostate cancer cells, with no accompanying augmentation of mRNA levels for the EMT transcription factors that regulate mesenchymal protein expression [89]. Moreover, exogenous androgens increase the expression of dynein, while pharmacological inhibition of dynein in castration-resistant prostate cancer cells promotes increased N-cadherin expression. This suggests that increased N-cadherin expression is possibly supported by a downregulation of retrograde trafficking machinery (Figure 3A) [89]. Thus, dysregulation of endosome trafficking appears to mediate altered expression and positioning of epithelial and mesenchymal proteins in prostate cancer to facilitate a partial EMT that promotes cancer metastasis.

Altered expression of integrin adhesion receptors and mesenchymal intermediate filaments, such as vimentin, are also important features of prostate cancer cells undergoing EMT and can potentiate cell migration to facilitate metastasis [90,91,92]. Notably, vimentin is increased in metastases and circulating tumour cells relative to primary well-differentiated tumours, suggesting that exploring the mechanistic interplay with the endosome-lysosome system could reveal selective therapeutic targets [91,93,94]. Overexpression of vimentin has been shown to promote the recycling of integrins and cell migration, suggesting that overexpression of vimentin in cancer cells may increase the endosomal recycling of migratory factors [95]. This concept is supported by the observation that decreased vimentin interferes with endosome localisation and the dynamic maturation and acidification of early endosomes, leading to endosomal enlargement and cargo arrest [96]. Vimentin interacts with and is phosphorylated by active Rab7, a mechanism that potentiates the rearrangement of the vimentin network [97,98]. Notably, this interaction happens during a Rab7 GDP to GTP switch, which recruits RILP and dynein trafficking machinery to promote Rab7 late endosome maturation to lysosomes [99,100]. Furthermore, vimentin also undergoes bi-directional movement on microtubules and appears to require dynein and kinesin regulation for precise cellular localisation [60,101,102]. This therefore acts as a potential mechanism for regulating cell migration, which requires the transport of vimentin to cell protrusions (Figure 3A). Vimentin positioning mediated by vesicular trafficking machinery, which in turn affects key endosome functions that regulate migratory factors, appears to be a critical determinant for cancer cell migration. A further understanding of the interplay between vimentin, vesicular trafficking machinery, and endosome transport could provide valuable insights into potential therapeutic targets that inhibit prostate cancer progression.

Prostate cancer cells exploit aberrant expression of integrin adhesion receptors, which mediate cell adherence to the ECM and generate tractional forces for cell migration, to support increased tumour cell detachment and directed migration during metastasis [103,104,105,106]. While most integrins exhibit decreased expression in prostate cancer compared to normal prostate tissue, some have increased expression, and others can change during cancer metastasis [107,108]. This plasticity in integrin expression may be achieved by dynamic modulation of endosomes and lysosomes and may play a role in promoting a shift from an inefficient random to a highly efficient persistent directional cancer cell migration, which requires organelle polarisation at cell protrusions [109]. Indeed, endosomal regulation of integrin cell surface expression requires both Rab4- and Rab11-positive endosomes [104,110]. Increased Rab4 endosomal recycling of the integrin αvβ3 and attenuated Rab11 endosomal recycling of α5β1 at migratory cell projections promotes persistent cell migration [104]. Conversely, increased Rab11 endosomal recycling of α5β1 and attenuated Rab4 endosomal recycling of αvβ3 promotes random migration, suggesting that differential activity and kinetics of particular recycling endosomes carrying specific integrins may affect the migration potential of cells [104]. The ability to selectively downregulate and upregulate Rab4 and Rab11 endosome recycling kinetics is likely mediated by trafficking machinery. The selective shift in balance between respectively fast and slow recycling depends on the expression of specific trafficking motor proteins. For example, reduced kinesin motor protein KIF16B promotes transferrin recycling, whilst concomitantly promoting EGFR degradation whereas overexpression of KIF16B has the opposite effect [51]. Therefore, plasticity to engage specific recycling endosome kinetics to modulate integrin-mediated cell adhesion may play a critical role in prostate cancer cell migration (Figure 3C).

Proteolysis of the extracellular matrix is required for prostate cancer cell migration and involves the secretion of proteolytic enzymes such as the matrix metalloproteinases (MMPs) MMP-2, 3, 7, 9, and cathepsins from late endosomes and lysosomes [8,111,112,113]. The increased activity of these proteases in prostate cancer cells may reflect concomitant upregulation of endosome anterograde trafficking, which promotes delivery of MMP-containing endosomes-lysosomes to the cell periphery, resulting in secretion or localisation on the cell surface (Figure 3D) [33]. Indeed, in prostate cancer cells, MMP secretion can be stimulated by hepatocyte growth factor, which has been shown to recruit kinesin machinery to promote endosome anterograde transport [22,112,114]. This may further be supported by inefficient recruitment of anterograde transport machinery and increased engagement of retrograde transport machinery, involving expression changes in adaptor proteins or endosome transmembrane proteins. This has been observed in invasive breast cancer cells and leads to the perinuclear arrest of late endosomes and decreased delivery of MMPs to invadopodia [33,115]. Similarly, increased secretion of proteinases may be mediated by mispositioning of lysosomes to the periphery of cancer cells, which can be induced by factors in the tumour microenvironment such as growth factors, acidic pH, and chemotherapeutic agents [116,117,118]. This plasticity of endosome-lysosome positioning in response to external factors may be mediated by vesicular trafficking machinery adaptor proteins with specific sensor capacity that recruit endosomes-lysosomes for microtubule-based transport depending on cellular context such as nutrient availability [119]. Of particular note is the lysosome transmembrane protein TMEM55B, which is upregulated by the transcription factor TFEB in response to cell starvation to promote dynein-mediated perinuclear clustering of lysosomes that in turn mediate autophagy [62]. TFEB is upregulated in prostate cancer and is thought to promote cancer progression by regulating lysosome biogenesis and plays a role in the resistance of cancer cells to the taxane docetaxel [118,120]. Lysosome trafficking machinery engaged downstream of TFEB, to mediate lysosome mispositioning, may therefore be critical targets for modulating the pro-metastatic effect of altered TFEB expression [118]. Thus, mispositioning of endosomes and lysosomes, mediated by altered trafficking machinery, may therefore enable cancer cells to proteolytically modify the ECM and respond to factors in the tumour microenvironment to support their metastatic dissemination.

During the initiation of metastasis, prostate cancer cells respond to a hypoxic microenvironment, which can be overcome by several mechanisms, including metabolic adaptations that may be mediated by alterations to endosome trafficking [121]. Indeed, prostate cancer cells induce hypoxia response transcription factors, such as HIF-1α, that upregulate endosome recycling of factors, such as glucose transporters that are crucial for metabolic adaptation [122,123]. Prostate cancer cells have increased levels of ATP relative to benign cells, which may affect the processivity of dynein and kinesin motors to support endosome recycling kinetics that are imperative for metabolic adaptation (Figure 3A) [124]. Indeed, kinesin and dynein processivity is increased by higher ATP concentrations, depending on the density of each motor protein recruited to cargo on microtubules [125,126,127,128]. Consequently, it is plausible that a metabolic switch from an efficient ATP-yielding glucose to an inefficient ATP-yielding lipid metabolism in prostate cancer, or vice versa, may involve alterations to endosome trafficking events that are mediated by kinesin and dynein. Consistent with this idea is the finding that prostate cancer cells that utilise glucose metabolism have an increased plasma membrane expression of glucose transporters, but in prostate cancer cells that utilise lipid metabolism, the glucose transporters are sequestered into endosomal pools [14]. This observation aligns with the possibility of increased kinesin-mediated endosome recycling of glucose transporters in an efficient ATP production metabolic state and increased dynein-mediated lysosome degradation in an inefficient ATP production metabolic state. However, a significant gap in our understanding is whether the copy number of dynein or kinesin subunits affects the density of the motor proteins for cargo that is recruited to microtubules or which adaptor proteins can alter the density to modulate the direction of bidirectional endosome transport in different metabolic states of the cell.

## 4. Tumour Cells Hijack Endosome/Cell-Surface Receptors and Signalling Cascades to Access and Migrate Through the Vasculature

**Figure 4:**
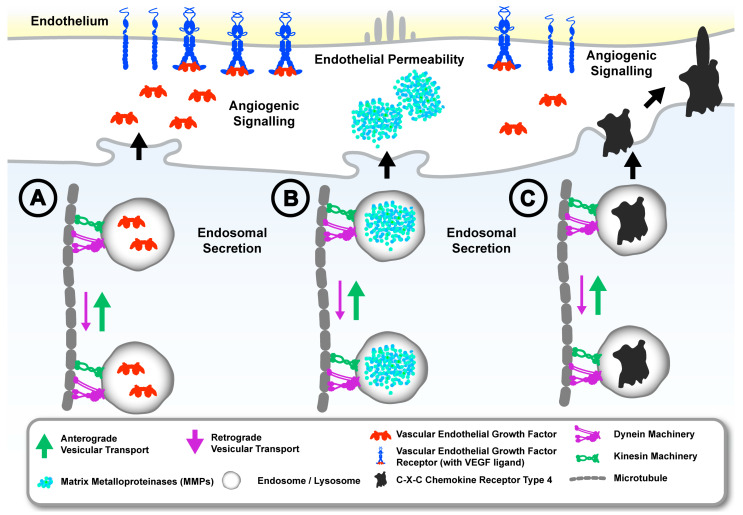
Dysregulated anterograde vesicular transport in prostate cancer cells may support secretion of factors and expression of receptors that promote interaction with the endothelium and the secretion of proteolytic factors that promote endothelial permeability. For example, (A), increased endosome secretion of proteins such as vascular endothelial growth factor, (B), secretion of proteinases, and (C), increased expression of receptors such as CXCR4 may be mediated by dysregulated anterograde trafficking machinery to support angiogenic signalling and endothelial permeability, which enables prostate cancer cells to intravasate and migrate into the circulation.

Dysregulation of endosome machinery can facilitate the modification of the local microenvironment during prostate cancer invasion and migration and may also be exploited for successful intravasation of prostate cancer cells during their metastatic dissemination. This may involve a capacity to directly interact with endothelial factors to promote angiogenesis and endothelial permeability. For instance, increased endosomal secretion of vascular endothelial growth factor (VEGF) by prostate cancer cells not only promotes angiogenesis but also increases endothelial permeability to enable cancer cell intravasation (Figure 4A,B) [129]. Interestingly, increased VEGF secretion from prostate cancer cells was mediated by increased exocytosis from Rab11 endosomes, which was supported by increased expression of the myosin VI motor protein that promotes exocytosis by tethering secretory cargo to the actin network [34,130]. Studies on hippocampal neurons have shown that the transport of VEGF-containing endosomes is predominantly anterograde and is disrupted by the knockdown of kinesin-1B [131]. Therefore, dysregulated anterograde endosome trafficking machinery may account for the increased secretion of VEGF in prostate cancer cells, in combination with factors such as myosin-mediated endosome tethering (Figure 4A). The increased expression of chemokines that are regulated by endosomes and lysosomes, such as CXCL8, CXCL12, CCL2, and CXCR4, in prostate cancer cells enables them to directly interact with endothelial cells to support intravasation [132,133]. It has been shown that the chemokines are mainly sorted for lysosomal degradation upon endocytosis, suggesting that their upregulation in prostate cancer may also reflect a downregulation of lysosome degradation kinetics (Figure 4C) [134]. Thus, the dysregulation of endosome trafficking machinery appears to be imperative for the endosomal mechanisms that enable the intravasation of prostate cancer cells. Further exploration of the specific adaptor proteins and motor proteins involved in this process is an important focus for further research on cancer metastasis.

## 5. Survival of Cancer Cells in the Circulation Is Dependent on the Evasion of the Immune System and Interactions with Endothelial Factors

**Figure 5:**
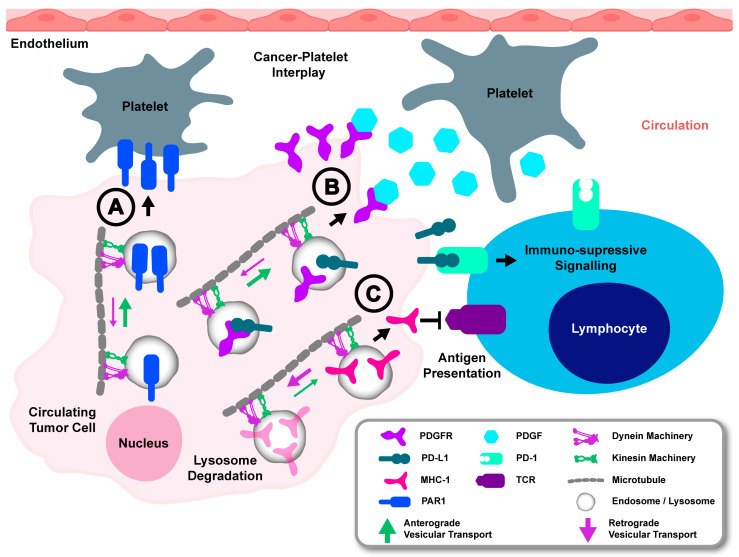
Altered endosome trafficking may support survival of prostate cancer circulating tumour cells. (A) Anterograde trafficking machinery may promote the trafficking of factors such as Protease-activated receptors 1 (PAR1) that enable interaction with platelets. (B) Altered endosome trafficking machinery may promote endosome recycling of receptors such as platelet-derived growth factor receptor (PDGFR), which promotes survival of circulating tumour cells by responding to growth factors secreted by platelets, such as platelet-derived growth factor (PDGF). This altered endosome recycling dynamics may also support the expression of programmed death-ligand 1 (PD-L1), which interacts with the programmed death 1 (PD-1) receptor on lymphocytes to propagate immune suppression signalling. (C) Retrograde trafficking of immune presentation factors such as major histocompatibility complex class I (MHC-I) molecules may contribute to cyto-protection of cancer cells against immune attack by lymphocytes potentially inhibiting binding to T-cell receptor (TCR).

Immune evasion and cytoprotective interactions with endothelial factors are mechanisms regulated by endosomes and lysosomes and may be utilised by prostate circulating tumour cells (CTCs) to support their survival in the circulation [135,136]. The downregulation of major histocompatibility complex (MHC) class I molecules by lysosome degradation to facilitate immune evasion may involve the recruitment of specific trafficking machinery that mediates endosome maturation to lysosomes (Figure 5C) [135,137]. It has been reported that late endosomes carrying MHC class II are retained in a perinuclear region when their dynein-mediated motility overpowers kinesin-mediated motility, and this attenuates MHC class II antigen presentation, implicating a requirement for vesicular trafficking machinery activity for the endosome regulation of MHC proteins [138]. MHC class I colocalises with MHC class II endosomes, suggesting that alterations to the transport of MHC class II endosomes may also affect MHC class I trafficking [139]. Indeed, KIF13A and KIF5B kinesin motors are key mediators of the endosome traffic that may promote recycling of MHC class I molecules, highlighting a role for kinesins in modulating antigen presentation in the immune system [48,140,141]. KIF5B is increased in prostate cancer cells, but whether it mediates the ability of prostate cancer CTCs to downregulate MHC class I as a mechanism for immune evasion has not been explored [47,48]. Prostate cancer cells also overexpress endosome-regulated ligands such as programmed death-ligand 1 (PD-L1), which enable direct interaction with T lymphocytes to suppress the immune system, suggesting that prostate cancer cells harbour the capacity for endosomal immunomodulatory plasticity to downregulate and upregulate pathways that mediate a net effect of immune suppression when interacting with lymphocytes (Figure 5B) [142]. Anti-tumour activity of immune checkpoint inhibitors, such as those directed against CTLA4, has demonstrated that enhancing the interactions of cancer cells with immune cells is a targetable mechanism for prostate cancer immunotherapy [143]. Thus, the potential role of endosome trafficking machinery to regulate endosomal immunomodulatory plasticity in prostate cancer cells presents an attractive target for intervention.

Impaired regulation of cell surface receptors by endosomes and lysosomes can lead to abnormal receptor expression and dysregulated endosome secretory function, which may support the survival of CTCs in the vasculature by facilitating interactions with endothelial factors and platelets. For example, prostate cancer cells derived from bone metastases overexpress protease-activated receptor 1 (PAR1), a thrombin receptor that enables CTCs to interact with platelets and to form protective heterotypic clusters (Figure 5A) [144,145]. Normally, PAR1 is quickly endocytosed and targeted for endosomal-lysosomal degradation upon activation by thrombin [146,147]. However, Rab11 endosomal recycling can maintain cell surface expression and signalling duration for both inactive and active PAR1 [146,147]. Interestingly, invasive breast cancer cells overexpress and maintain the activation of PAR1 even without thrombin, possibly due to increased recycling of PAR1 and failure to sort it into degradative endosomes-lysosomes [146]. This upregulation of PAR1 in cancer cells is typically seen in advanced stages of metastasis, where endosomes and lysosomes also show altered localisation and function, suggesting dysfunction in vesicular trafficking machinery may be involved in mediating this endosome positioning and function (Figure 5) [117,144,146]. The dysregulation of endosome secretion of metalloproteinases due to altered trafficking machinery may also function in CTCs to support their interactions with platelets. Consequently, upregulated MMP-2 secretion by metastatic prostate cancer cells can promote platelet aggregation [148]. Moreover, the secretion of growth factors by platelets, such as platelet-derived growth factor, may help promote the survival of prostate cancer circulating tumour cells. This may operate by sustained endosomal recycling of receptors that are receptive to these growth factors [149,150]. In this context, the activation of platelet-derived growth factor receptor is known to mediate the interaction with phosphatidylinositol 3-kinase, which promotes kinesin-mediated anterograde transport of recycling endosomes [150,151]. This suggests a role for endosome trafficking machinery in promoting processes that are imperative for the survival of CTCs. Alterations to the vesicular trafficking machinery that mediates the endosomal upregulation of factors, which facilitate cancer cell interactions with endothelial factors, may therefore be a critical mechanism that supports the survival and metastasis of cancer cells.

## 6. Invadopodium Formation, Aberrant Receptor Expression, and Protein Secretion Support the Extravasation, Dormancy, and Colonisation of Metastatic Cancer Cells

**Figure 6:**
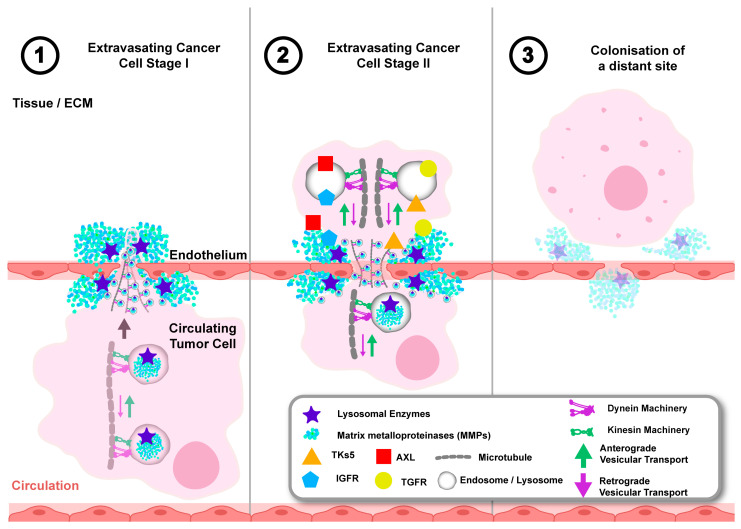
Trafficking of endosomes and lysosomes to the cell periphery may support the stages of extravasation and survival of prostate cancer cells at distant sites. During stages 1–2, dysregulated anterograde and retrograde trafficking mediated by kinesin and dynein trafficking machinery may be involved in the recruitment of endosomes carrying proteins such as tyrosine kinase substrate 5 (Tks5) and serine/threonine-protein kinase 3 (TAO3) to invadopodia projections to support the secretion of metalloproteinases (MMPs) to proteolytically modify the endothelium at sites of metastasis. During stages 2–3, dysregulation of trafficking machinery that results in anterograde trafficking of endosomes may contribute to mispositioning that supports secretion of proteins such as bone morphogenic proteins (BMP), parathyroid hormone-related protein (PTHrP), and expression of receptors such as AXL, transforming growth factor receptor (TGFR), and insulin growth factor receptor (IGFR) that integrate signalling cascades to enable interactions with resident cells in the site of metastasis and enable proteolytic modification of the extracellular matrix (ECM) at the site of metastasis.

Prostate cancer CTCs form invadopodium projections, which are enriched with endosome-regulated proteins such as the metalloproteinase MT1-MMP and tyrosine kinase substrate 5 (Tks5), and they can utilise these to proteolytically modify the endothelium and gain access to distant sites (Figure 6) [152,153,154,155]. Tks5 is an important scaffolding protein that requires regulation by the serine/threonine-protein kinase 3 (TAO3) and endosome transport for incorporation in invadopodia [155,156,157]. Interestingly, TAO3 promoted invadopodium formation by promoting Tks5 trafficking in recycling endosomes, involving inhibiting the activation of the dynein light chain LIC2, suggesting that recycling endosome anterograde kinetics may supersede retrograde kinetics to mediate invadopodium formation and to potentiate extravasation [157]. Moreover, Rab40b is an important Tks5 binding partner that localises to recycling endosomes, and its upregulation in cancer cells to promote MMP secretion and invadopodium-mediated proteolysis of the ECM [158]. Rab40b also localises to the leading edge of migrating cells, where it interacts with Rap2 GTPase, a small GTPase required for cell migration, preventing its lysosomal degradation and recycling back to migratory projections. This further highlights the exploitation of endosome trafficking kinetics in pathways that regulate invadopodium formation to mediate cell migration [159]. Altered endosome spatial and temporal kinetics may play a critical role in invadopodium formation, proteolysis of the ECM, and cancer cell migration, which are required for the successful extravasation of prostate cancer cells.

Tumour cells disseminating to a distant site may attenuate their response to therapy and evade clinical detection by remaining dormant until conditions favour proliferation and formation of a metastatic tumour [160]. Indeed, disseminated prostate cancer cells (DTCs) that have gained access to the bone marrow can remain dormant by exploiting quiescent cell signalling, which is usually reserved for the regulation of haemopoietic stem cells [160,161]. DTCs may exhibit dysregulated receptor expression that enables them to colonise the haematopoietic stem cell niche and respond to ligands secreted by resident osteoblasts and endothelial cells that can induce DTC dormancy [162]. For example, AXL is an important receptor tyrosine kinase that is expressed by prostate cancer DTCs and responds to growth arrest-specific 6 (Gas6) protein secreted by osteoblasts and promotes dormancy by limiting DTC proliferation (Figure 6) [163]. Regulation of AXL by the endosome-lysosome system is thought to be initiated when AXL is endocytosed upon Gas6 stimulation, whereby it is either recycled back to the plasma membrane or targeted to endosomes-lysosomes for degradation [164]. Under hypoxic conditions, increased AXL expression and signalling is independent of Gas6, probably due to its delayed endosomal-lysosomal degradation, and this may also promote prolonged signalling (Figure 6) [164,165]. Interestingly, overexpression of the kinesin motor protein KIF16B can inhibit the endosomal-lysosomal degradation of EGFR, highlighting that the degradation kinetics are directly affected by alterations to specific vesicular trafficking machinery [51]. Moreover, altered expression of several kinesins has been described to play a role in prostate cancer metastasis by promoting aberrant AR signalling and inhibiting apoptosis, but their role in regulating endosome-lysosome degradation dynamics remain relatively unexplored [166,167]. The attenuated endosomal-lysosomal degradation of aberrantly expressed receptors may be a key mechanism used by DTCs to prolong signalling cascades. This would support their dormancy at a secondary site, and the potential role of vesicular trafficking machinery in this process warrants further investigation.

Prostate cancer DTCs utilise signalling from growth factors such as transforming growth factor beta (TGF-β) and insulin-like growth factor 1 (IGF-1), which are liberated by osteoblastic and osteoclastic remodelling of the bone, to support their proliferation, survival, and subsequent colonisation [162,168,169]. To induce osteoclast and osteoblast bone remodelling, prostate cancer cells secrete endosome-regulated factors such as parathyroid hormone-related protein (PTHrP) and bone morphogenic proteins (BMPs) [170]. Both BMP and PTHrP secretion require trafficking by Rab11 endosomes and altered fusion with the plasma membrane; hence the upregulation of Rab11 in prostate cancer may facilitate increased secretion of these factors for bone remodelling [171,172]. The sorting of cargo to Rab11 recycling endosomes is dependent on kinesin motors, which promotes increased peripheral trafficking and exocytosis of cargo from recycling endosomes [50,173]. Indeed, kinesin-1 and kinesin-3 trafficking machinery have been shown to potentiate endosome exocytosis, while increased Rab11 endosomes in prostate cancer provide circumstantial evidence of potential alterations to recycling endosome dynamics [174,175,176,177,178,179]. Further characterisation of the role of vesicular trafficking machinery is required to determine if this is critical for endosomal secretion of bone resorption factors to support the colonisation and survival of prostate cancer cells.

Extracellular vesicles (EVs) are crucial for intercellular communication and are released by prostate cancer cells to aid in interactions with the extracellular matrix and to modulate the immune response. EVs are therefore able to influence therapeutic outcomes and to support cancer cell dissemination [180,181]. Emerging evidence suggests that the contents of prostate cancer-derived EVs, such as miRNAs, proteins, and lipids, may also serve as biomarkers for disease diagnosis and prognosis, highlighting important clinical potential [182,183,184,185]. The fusion of multivesicular bodies with the plasma membrane is an important mechanism of EV release and may be affected by changes in endosome trafficking dynamics [186]. Indeed, the upregulation of the kinesin motor KIFC2 in prostate cancer is known to regulate the transport of multivesicular bodies [41,42]. Rab27 is one of the critical Rab GTPases that regulates the release of EVs and is also upregulated in prostate cancer [187,188]. Interestingly, the ability of Rab27 to promote exosome release is regulated by the scaffolding protein KIBRA, which is also upregulated in prostate cancer and requires interaction with dynein for its co-activator functions [189,190,191]. Consequently, the altered endosome biology that promotes increased EV release by prostate cancer cells involves utilising specific components of the vesicular trafficking machinery.

## 7. The Potential for Altered Vesicular Trafficking Machinery to Inform on Prostate Cancer Diagnosis and Prognosis

**Figure 7:**
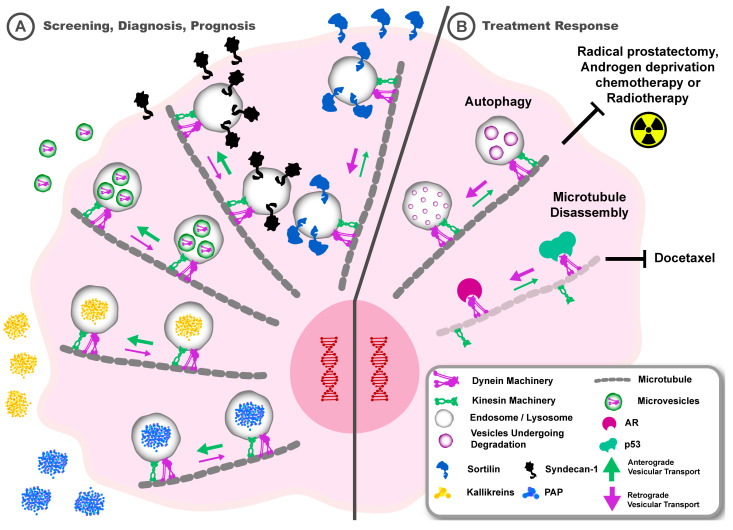
Alterations to vesicular trafficking machinery may have implications for the screening, diagnosis, prognosis, and treatment response for prostate cancer. (A) Anterograde vesicular trafficking machinery may operate to promote endosomal secretion of biomarkers such as kallikreins, prostatic acid phosphatase, and secretion of microvesicles enriched with vesicular trafficking machinery proteins. Upregulation of syndecan-1 and reduction in sortilin in advanced prostate cancer is associated with defects in endosome trafficking that may involve vesicular trafficking machinery. (B) Germline and somatic mutations may directly affect vesicular trafficking machinery or indirectly through promoting vesicular trafficking machinery-regulated endosomal pathways such as autophagy that facilitate poor treatment response for prostate cancer. Microtubule disassembly mediated by kinesin motor proteins may operate as a mechanism of resistance for agents such as docetaxel. Dynein motor proteins may be involved in aberrant translocation transcription factors such as androgen receptor (AR) and tumour suppressors such as p53 to the nucleus.

There is an urgent need for more effective biomarkers to improve risk stratification in the management of patients with prostate cancer and particularly to accurately assign patients to either active surveillance or specific interventions [192]. The kallikrein panel, consisting of the endosomal secreted proteins total PSA, free PSA, intact PSA, and human kallikrein-2, is the current gold standard screening approach for prostate cancer but was originally only proposed to be used for monitoring [193]. PSA colocalises with motor protein myosin VI in transferrin-positive recycling endosomes in prostate cancer cells, and its secretion was reduced when myosin VI was knocked down, highlighting a critical role for endosome transport in modulating PSA secretion [34]. Interestingly, myosin VI is overexpressed in prostate cancer, with the highest expression in high-grade prostate cancers, suggesting that the motor proteins regulating endosome transport and secretion of kallikrein biomarkers may help inform on advanced prostate cancer [194]. It is likely that bidirectional endosome transport on microtubules may also impact the secretion of kallikreins, as kinesin and dynein motor proteins are required for the transport of transferrin-positive recycling endosomes, and changes in their expression are sufficient to modulate endosome trafficking (Figure 7A) [51,195]. Proteomic analysis of microvesicles secreted by prostate cancer cells revealed a high content of cytoskeletal proteins, including the heavy chain of dynein, suggesting a potential avenue for the clinical detection of aberrantly expressed vesicular trafficking machinery that may inform on prostate cancer prognosis [196].

Assessment of prostate histopathology using H&E staining, which is often complemented by immunohistochemistry, remains the gold standard for prostate cancer diagnosis [197]. Upregulation of biomarkers, including PSA, prostatic acid phosphatase, and prostein, in prostate cancer may involve altered endosome trafficking kinetics that regulate protein localisation, degradation, and secretion, and is likely controlled by changes in vesicular trafficking machinery (Figure 7A) [34,35,197]. Direct examination of the endosome-lysosome system revealed that there is increased syndecan-1 and reduced sortilin in high-grade prostate cancer, and mechanistic studies further linked the activity of these proteins to dysregulated endosome trafficking and a lipogenic phenotype associated with a higher metastatic propensity [8,14]. Altered vesicular trafficking machinery may play a critical role in the aberrant protein expression, location, and function of these endosome-regulated biomarkers. For instance, sortilin interacts with early and recycling endosomes (positive for Rabs 4, 10, 11, and 14) to regulate glucose transporter 1 transport in androgen-responsive prostate cancer cells [14,198,199,200]. Syndecan-1, when overexpressed in advanced prostate cancer cells, promotes an invasive lipogenic phenotype by interaction with β3 integrin, facilitated by concurrent expression of MT1-MMP [14]. Additionally, prostatic acid phosphatase relies on trafficking within Rab27 endosomes for secretion [35]. Earlier we described a model for altered vesicular trafficking machinery that contributes to dysfunction in endosomal recycling and secretion during prostate cancer metastasis and which impacts the function of proteins implicated in prostate cancer metastasis. This highlights the potential opportunity to explore vesicular trafficking machinery biology further for biomarker discovery in prostate cancer.

## 8. Altered Vesicular Trafficking Machinery May Correlate with Poorer Outcomes for Patients with Prostate Cancer

Vesicular trafficking machinery requires microtubules to transport endosomal cargo and would therefore be affected by chemotherapeutic agents that stabilise these microtubules. Indeed, prostate cancer chemotherapeutics such as docetaxel function by stabilising microtubules, leading to mitotic arrest and apoptosis of cancer cells, in addition to inhibiting translocation of the oncogenic androgen receptor (AR) to the nucleus [201,202]. It has been postulated that overexpression of kinesins in the kinesin 1, 5, 12, and 14 families may facilitate docetaxel resistance by binding to and altering microtubule stabilisation (Figure 6B) [203,204,205,206]. Moreover, activation of EMT and autophagy, which we highlighted as being regulated by vesicular transport machinery, are recognised as mechanisms for docetaxel resistance that complicate treatment outcomes [207,208]. Several other lines of evidence point to a critical role of trafficking machinery in modulating treatment response. For instance, breast cancer cells that were resistant to ionising radiation engaged the kinesin adaptor protein Arl8b to recruit lysosomes for anterograde transport and subsequent proteinase secretion promoted cancer cell invasion into the ECM [56]. This suggests that aberrant trafficking of endosomes and lysosomes may contribute to radiotherapy resistance, a concept that needs further investigation in prostate cancer [209,210]. The ability of dynein and kinesin motor proteins to modulate the stability of microtubules by mediating the assembly and disassembly of tubulin proteins may also have direct consequences for treatment response [211]. Indeed, decreased dynein expression in patient tissues with colorectal cancer was found to be associated with poor outcome and was postulated to relate to microtubule dynamics, possibly by enabling kinesin-mediated microtubule disassembly to induce a phenotype that is resistant to therapy [212]. Furthermore, whilst colorectal cancer cells are sensitised to therapeutic agents such as oxaliplatin in the absence of dynein, taxanes that stabilise the microtubule are ineffective, possibly due to increased kinesin-promoted disassembly (Figure 7B) [57,213]. Thus, altered trafficking machinery-mediated transport of endosomes and lysosomes may persist in cancer cells and dictate response to therapy. Proof of concept for the therapeutic targeting of trafficking machinery motor proteins in cancer has been demonstrated with the discovery of the aminothiazole dynarrestin, a novel inhibitor of dynein that inhibited the proliferation of oesophageal squamous cell carcinoma cells [214]. Furthermore, the potential for targeting kinesins in cancer has also been reviewed, though the current compounds developed target kinesins predominantly involved in regulating mitosis and cytokinesis, and not those involved in regulating endosome dynamics during cancer metastasis [215,216,217].As dysfunction of vesicular trafficking machinery affects specific endosome pathways in the metastatic cascade, targeting the machinery that is recruited to mediate specific endosome functions may be attractive in precision medicine applications for the clinical management of prostate cancer.

Chimeric antigen receptor T-cell (CAR-T) therapy and immune checkpoint inhibitors are emerging as promising therapeutic approaches for prostate cancer and rely on targeting increased expression of antigens on prostate cancer cells, such as prostate-specific membrane antigen (PSMA), or modulating the expression of increased immunosuppressive receptors, such as PD-L1, respectively [142,218,219]. Challenges with clinical efficacy of these approaches include the immunosuppressive microenvironment, which involves vesicular trafficking to dynamically modulate the surface expression of receptors and antigens to facilitate prostate cancer cell immune escape during metastasis [220,221]. Targeting the vesicular trafficking machinery to control expression of immunomodulatory factors for therapeutic benefit is compelling and is supported by the observation that inhibition of the endosome trafficking regulator neuropilin-2 reduces PD-L1 expression and enables antitumor immune cell activation in prostate cancer cells [63,64].

DNA sequencing for oncogenic mutations that are frequently detected in prostate cancer metastasis is an important approach for understanding prostate cancer disease predisposition, progression, and, more increasingly, for monitoring clinical outcomes. This is supported by recent advances in sequencing technology and knowledge of specific germline and somatic mutations involved in prostate cancer metastasis [222,223]. Mutations in vesicular trafficking machinery genes or known oncogenes could potentially disrupt endosome-lysosome function, which may disrupt or promote a metastatic phenotype in prostate cancer cells. In the former case, mutations in motor proteins may impair their processivity and ability to engage adaptor proteins and recruit cargo for microtubule-based transport [224,225]. In the latter case, germline and somatic mutations in known oncogenes may promote cell adaptation mechanisms that involve engagement and disruption of vesicular trafficking systems. Analysis of the prostate cancer exome revealed that the kinesin heavy chain gene KIF5A was mutated in some prostate cancers, and amplification of KIF18B was significantly higher in tumours with PTEN mutations, supporting the hypotheses that vesicular trafficking machinery mutations may be oncogenic and that oncogenic mutations may involve vesicular trafficking machinery (Figure 7B) [166,226]. Specific mutations have significant implications for treatment response in prostate cancer. For instance, germline mutations such as BRAC1/BRAC2 and somatic mutations such as tumour protein p53 (p53) are notably associated with increased risk of prostate cancer progression and poor survival outcomes following treatments such as radical prostatectomy, androgen deprivation chemotherapy, or radiotherapy [223,227,228,229,230,231]. Inducing autophagy, a process regulated by endosomes and lysosomes, is one of the mechanisms for treatment resistance, and this is affected by these mutations. This suggests that engagement of the endosome-lysosome system downstream of the oncogenic mutations could be a potential mechanism for how these mutations potentiate poor treatment response in prostate cancer (Figure 7B) [232,233,234]. However, it is important to acknowledge that while we highlight specific mutations, there are other mutations in prostate cancer that also impact treatment response [222,227]. These mutations in prostate cancer warrant further investigation to understand how they may modulate spatial-temporal dynamics in the endosome-lysosome system as a potential mechanism of therapy resistance. p53 mutations are particularly interesting as p53 is transported to the nucleus by dynein motor proteins, and it would be interesting to explore how the germline mutations in p53 change this interaction [235]. Moreover, p53 mutants have been shown to promote amplification of the endosome-related protein dynamin, which is involved in the recruitment of APPL1 early endosome recycling machinery and increased recycling of EGFR and β-integrin, which promotes migration and invasion of non-small cell lung cancer cells [236]. In prostate cancer cells with p53 mutations, a similar mechanism may operate, as these cells exhibit high expression and pro-migratory signalling of EGFR and altered expression of the APPL1 protein, consistent with increased endosomal recycling [8,237,238]. This may have significant clinical significance, as inhibiting EGFR signalling can sensitise prostate cancer cells to chemotherapeutic agents such as Adriamycin [239]. Furthermore, mutant p53 promotes the activation of ARF6, which in turn functions to modulate endosome recycling kinetics by facilitating the switch between dynein and kinesin motor proteins [33,240]. We have described the role of dysfunctional trafficking machinery in mediating changes in vesicular transport that may be required for autophagy and endosome recycling to promote prostate cancer metastasis. It is therefore conceivable that specific combinations of germline and somatic mutations may promote prostate cancer metastasis and poor treatment response by manipulating this vesicular trafficking machinery to control the function of the endosome-lysosome system (Figure 7).

Clinical trials have demonstrated that benefits in overall survival can be derived from the combination therapy of docetaxel and next-generation anti-androgens such as enzalutamide [241]. Although there is little or no clinical data on correcting endosome dysfunction by directly targeting vesicular trafficking machinery in metastatic prostate cancer, current therapeutics that target endosome function show the potential of synergising with androgen deprivation compounds. For instance, inhibiting endosome recycling by the anti-malarial small molecule primaquine in prostate cancer cells reduced the expression of androgen receptor, modulated lysosome degradation kinetics, and resulted in decreased cell survival independently. This effect was greater when used in combination with enzalutamide, demonstrating the viability of targeting recycling endosome function, which we contend contributes to prostate cancer metastasis and is modulated by vesicular trafficking machinery [242]. Drug discovery efforts focusing on modifying the vesicular trafficking machinery may provide a novel therapeutic strategy for metastatic prostate cancer to improve patient outcomes.

## 9. Conclusions

An increased understanding of the clinical significance of the altered expression of specific vesicular trafficking machinery during metastasis could potentially identify novel markers for prostate cancer management and intervention. Circumstantial evidence points to the potential role of adaptor proteins in prostate cancer progression. However, a comprehensive investigation into endosome-lysosome recruitment and interaction with specific motor proteins, plus the involvement in mediating particular events in the prostate cancer metastatic cascade, is yet to be completed. For example, the adaptor protein JIP4, which regulates the endosomal trafficking of metalloproteinases during cancer cell migration, is increased in prostate cancer tissue and is associated with poor prognosis for prostate cancer patients [38]. Moreover, the adaptor protein Hook3, which interacts with both the dynein and kinesin motor proteins to recruit early endosomes for retrograde transport, is overexpressed in prostate cancer and has been associated with poor prognosis [59,243]. The observed alterations in early endosome biogenesis and trafficking in prostate cancer, along with the potential involvement of alterations in the vesicular trafficking machinery, underscore the need for further research into the diagnostic and prognostic potential of this cellular machinery. Our discussion also highlights the potential for targeting the dynein and kinesin trafficking machinery for therapeutic intervention in patients with prostate cancer. This could be a promising strategy for treating metastatic prostate cancer, especially when resistance is encountered with androgen-related therapies that target microtubules and AR nuclear translocation and poor efficacy of novel immunomodulatory approaches such as CAR-T cells and immune checkpoint inhibitors. Endosome-lysosome proteins can report on the primary pathogenesis in prostate cancer, and the vesicular trafficking machinery is involved in regulating critical events like microtubule polymerisation. This machinery can also alter the sensitivity of cancer cells to specific drugs based on motor protein expression. Therefore, a deeper understanding together with the potential to manipulate these mechanisms could pave the way for more effective diagnostics and treatments for patients with prostate cancer.

## Figures and Tables

**Table 1:** Microtubule vesicular trafficking machinery in prostate cancer metastasis: highlight of known and theoretical components.

Mode of Transport	Vesicular Trafficking Machinery	Component	Endosome Compartment	Role in PCa Metastasis	Refs.
Anterograde	Regulatory protein	ARF6	Lysosomes & Multivesicular bodies	Promotes cancer cell proliferation and invasion & May regulate extracellular vesicle release	[37,38,39,40]
	Motor protein	KIFC2	Multivesicular bodies	May regulate extracellular vesicle release	[41,42]
	Motor protein	KIF3A	Recycling endosomes	May regulate invasion and migration	[43,44]
	Motor protein	KIF3C	Recycling endosomes	May regulate treatment response	[45,46]
	Motor protein	KIF5B	Lysosomes	Promotes autophagy	[47]
	Motor protein	KIF5B	Early endosomes	May modulate immune responses	[48]
	Motor protein	KIF13A	Recycling endosomes	May regulate EMT	[43,49,50]
	Motor protein	KIF16B	Recycling endosomes	May regulate migration and invasion	[51,52]
	Motor protein	KLC1/2	Endosomes/ lysosomes	May regulate EMT and treatment response	[53,54,55]
	Adaptor protein	ARL8B	Lysosomes	May modulate treatment response	[53,56]
Retrograde	Adaptor protein	JIP3/4	Lysosomes	Promotes cancer cell migration and invasion	[38,53]
	Motor protein	DYNC1I1	Endosomes/ lysosomes	Modulates cancer cell migration and regulates EMT May modulate treatment response	[53,57,58]
	Adaptor protein	Hook3	Early endosomes	Associated with poor prognosis and may promote cancer cell proliferation	[59]
	Regulatory protein	NDEL1	Late endosomes	May regulate EMT	[60]
	Adaptor protein	RILP	Late endosomes/ lysosomes	Modulates cancer cell proliferation, migration, and invasion	[61]
	Adaptor protein	TMEM55B	Lysosomes	Promotes autophagy	[62]
	Adaptor protein	Neuropilin-2	Early endosomes	May modulate autophagy and immune response	[63,64,65]
	Regulatory protein	NDE1	Endosomes/ lysosomes	May regulate migration and invasion	[66]

## Abbreviations

ARF6	ADP-ribosylation factor 6
BMP	Bone morphogenic protein
CTC	Circulating tumour cell
DCT	Disseminated tumour cell
ECM	Extracellular matrix
EGFR	Epidermal growth factor receptor
EMT	Epithelial to mesenchymal transition
Gas6	Growth arrest-specific 6
IGF-1	Insulin-like growth factor 1
JIP3	JNK-interacting protein 3
JIP4	JNK-interacting protein 4
MHC	Major histocompatibility complex
MMP	Matrix metalloproteinase
PAR1	Protease-activated receptor 1
PD-L1	Programmed death-ligand 1
PSA	Prostate specific antigen
PTHrP	Parathyroid hormone-related protein
RILP	Rab interacting lysosomal protein
TAO3	serine/threonine-protein kinase 3
TGF-β	Transforming growth factor beta
Tks5	Tyrosine kinase substrate 5
VEGF	Vascular endothelial growth factor
VEGFR	Vascular endothelial growth factor receptor
